# From bench to bedside: the history and progress of CAR T cell therapy

**DOI:** 10.3389/fimmu.2023.1188049

**Published:** 2023-05-15

**Authors:** Aroshi Mitra, Amrita Barua, Luping Huang, Siddhartha Ganguly, Qin Feng, Bin He

**Affiliations:** ^1^ Center for Nuclear Receptors and Cell Signaling, Department of Biology and Biochemistry, University of Houston, Houston, TX, United States; ^2^ Immunobiology and Transplant Science Center, Departments of Surgery and Urology, Houston Methodist Research Institute, Houston Methodist Hospital, Houston, TX, United States; ^3^ Department of Medicine, Weill Cornell Medicine, Cornell University, New York, NY, United States; ^4^ Section of Hematology, Houston Methodist Neal Cancer Center, Houston Methodist Hospital, Houston, TX, United States

**Keywords:** chimeric antigen receptor (CAR T), cancer immunotherapy, tumor burden, cytokine release syndrome, TCR - T cell receptor

## Abstract

Chimeric antigen receptor (CAR) T cell therapy represents a major breakthrough in cancer care since the approval of tisagenlecleucel by the Food and Drug Administration in 2017 for the treatment of pediatric and young adult patients with relapsed or refractory acute lymphocytic leukemia. As of April 2023, six CAR T cell therapies have been approved, demonstrating unprecedented efficacy in patients with B-cell malignancies and multiple myeloma. However, adverse events such as cytokine release syndrome and immune effector cell-associated neurotoxicity pose significant challenges to CAR T cell therapy. The severity of these adverse events correlates with the pretreatment tumor burden, where a higher tumor burden results in more severe consequences. This observation is supported by the application of CD19-targeted CAR T cell therapy in autoimmune diseases including systemic lupus erythematosus and antisynthetase syndrome. These results indicate that initiating CAR T cell therapy early at low tumor burden or using debulking strategy prior to CAR T cell infusion may reduce the severity of adverse events. In addition, CAR T cell therapy is expensive and has limited effectiveness against solid tumors. In this article, we review the critical steps that led to this groundbreaking therapy and explore ongoing efforts to overcome these challenges. With the promise of more effective and safer CAR T cell therapies in development, we are optimistic that a broader range of cancer patients will benefit from this revolutionary therapy in the foreseeable future.

## 1 History of cancer immunotherapy

Immunotherapy has transformed cancer treatment, offering a beacon of hope to once-desperate patients with late-stage metastatic cancers. Science magazine recognized its impact by naming it the “Breakthrough of the Year” in 2013. The origins of this paradigm-shifting therapy can be traced back to the 1860s, when German physicians Wilhelm Busch and Friedrich Fehleisen independently observed tumor regression in patients infected with erysipelas (1). In the 1890s, Dr. William B. Coley, a bone surgeon and cancer researcher at New York Hospital (now part of New York-Presbyterian/Weill Cornell Medical Center), made similar observations and began injecting live bacteria into patients with inoperable malignant tumors. His treatment, known as “Coley’s Toxins”, achieved complete regression in many of the approximately one thousand cancer patients he treated (2). Today, Dr. Coley is hailed as the “Father of Cancer Immunotherapy” for his pioneering work. However, the use of Coley’s Toxins as a cancer treatment declined in the 1940s and was largely discontinued by the 1960s due to concerns about infectious agents, a lack of understanding of their mechanisms of action, and the emergence of radiation therapy and chemotherapy as alternative treatments. Despite this setback, the legacy of Dr. Coley’s work lives on in the ongoing development of immunotherapies that harness the power of the immune system to fight cancer.

In 1928, American biologist Raymond Pearl, working at Johns Hopkins Hospital in Baltimore, reported that cancer incidence was significantly lower in patients infected with Mycobacterium tuberculosis, based on a study of autopsies(3). This finding ignited interest in using an attenuated live bacterial vaccine, Bacillus Calmette-Guérin (BCG), as a cancer treatment. In 1976, the efficacy of intracavitary BCG in treating superficial bladder tumors in human patients was first reported(4), paving the way for the FDA’s approval of the use of the intravesical BCG vaccine for early-stage bladder cancer in 1990. Today, BCG immunotherapy remains a standard of care for high-risk non-muscle invasive bladder cancer and is believed to work by activating the immune system to attack cancer cells (5).

Interferons are a class of cytokine proteins that cells produce in response to viral infections or other stimuli. They were first discovered in the 1950s, and their antiviral and immune-regulatory properties were soon recognized. In the late 1960s, researchers discovered that interferons could suppress tumor growth in animals (6), and subsequent human clinical trials confirmed their anticancer activity (7–9). In 1986, IFNα became the first FDA-approved cancer immunotherapy when it was cleared for use in treating hairy-cell leukemia. Since then, interferons have been investigated for use in other types of cancer, with mixed results. While they can induce tumor cell death and stimulate immune responses, they can also have significant side effects, such as flu-like symptoms and depression (10). Despite these challenges, interferons remain an important part of the cancer immunotherapy arsenal and are being explored in combination with other immunotherapeutic agents to enhance their efficacy.

Another cytokine that has shown promising anticancer activity is interleukin-2 (IL-2), which was identified in 1976 as a T cell growth factor and its cDNA was cloned in 1983 (11). In the 1980s, Dr. Steven Rosenberg at the National Cancer Institute (NCI) conducted clinical trials in which they administered high doses of IL-2 to patients with advanced cancer, leading to partial or even complete remission of their tumors (12). While high-dose IL-2 therapy has an overall response rate of 15% in melanoma and kidney cancer patients (13, 14), it can also cause significant systemic toxicity known as vascular leak syndrome. The FDA approved the use of high-dose IL-2 In 1992 for the treatment of metastatic renal cell carcinoma, and in 1998 for metastatic melanoma. Today, IL-2 is still being utilized in certain cases to treat advanced melanoma and kidney cancer, particularly in combination with other immunotherapeutic agents to enhance their efficacy. Furthermore, IL-2 plays a critical role in priming tumor-infiltrating lymphocytes for the treatment of cancers such as metastatic melanoma and non-small cell lung cancer (15, 16).

IL-2 exerts its anticancer activity by binding to IL-2 receptors and promoting the proliferation of tumor-reactive T cells, which can specifically target and attack tumors in the body. The earliest evidence for the anticancer potential of T cells came from allogenic hematopoietic cell transplantation (HCT) (17), which has proven effective for treating patients with leukemia and other hematologic malignancies (18). The donor T cells in HCT can recognize and eliminate allogenic cancer cells in the patients, a phenomenon known as “graft-versus-tumor” (GVT) activity.

The immune checkpoint therapies of anti-PD-1, anti-PD-L1, and anti-CTLA-4 target inhibitory receptors expressed on activated T cells, for which Drs. James P. Allison and Tasuku Honjo were awarded the Nobel Prize in Physiology or Medicine in 2018. By blocking the PD-1 and PD-L1 interaction using antibodies, such as pembrolizumab and nivolumab, exhausted T cells are stimulated, and antitumor immunity is enhanced. The humanized IgG4 anti-PD-1 antibodies that are FDA-approved do not induce Fc-dependent cytotoxicity activity, suggesting that the simple blocking of the interaction between PD-1 and PD-L1 is sufficient to activate tumor-reactive T cells. These therapies have shown remarkable efficacy in treating several types of cancers, including melanoma, non-small cell lung cancer, and renal cell carcinoma.

The mechanism of anticancer action of the anti-CTLA-4 antibodies is more complex than that of anti-PD-1 antibodies, as CTLA-4 is highly expressed not only in activated T cells but also in regulatory T cells (Tregs). While hamster and mouse anti-mouse CTLA-4 antibodies were able to deplete intratumoral CTLA-4^+^ Treg cells in a mouse study (19), evidence suggests that ipilimumab, a humanized anti-CTLA-4 IgG1, may not have the same effect in human tumors (20). On the other hand, tremelimumab, another anti-CTLA-4 antibody, is a humanized IgG2 and lacks depletion activity. In November 2022, the FDA approved tremelimumab in combination with durvalumab (anti-PD-1) and platinum-based chemotherapy for the treatment of metastatic non-small cell lung cancer (NSCLC). Therefore, the anticancer activity of anti-CTLA-4 antibodies may involve both blocking CTLA-4 on activated T cells and depleting CTLA-4-expressing Treg cells.

It is worth noting that immune checkpoint inhibitors operate through a different mechanism when compared to traditional anticancer antibodies such as anti-CD20 rituximab and anti-Her2 trastuzumab. While the former activates tumor-reactive T cells, rituximab and trastuzumab largely rely on their Fc domain to recruit natural killer cells, macrophages, and the complement system to destroy cancer cells (21).

Adoptive transfer therapy using tumor-infiltrating lymphocytes (TILs) is another form of cancer immunotherapy developed by Dr. Steven Rosenberg at NCI (22–25). This approach involves harvesting of TILs from freshly resected tumor tissues, followed by their expansion in the laboratory, and then re-infusing them into patients. Although this therapy has achieved remarkable results in some patients with solid tumors, including objective and even complete remissions, there are several limitations associated with the adoptive transfer of TILs. These include the technical difficulties in isolating and expanding TILs, the high costs associated with personalized therapy, high variability in response, and limited applicability.

The effectiveness of high-dose IL-2 therapy, immune checkpoint inhibitors, and TILs in treating cancer relies on the presence of pre-existing tumor-reactive T cells in patients. However, due to immunoediting (26), only a small percentage of the non-synonymous mutations identified in tumors are immunogenic - typically less than 2% (27). Consequently, many tumors may not be immunogenic enough to trigger an immune response. Thus, the overall response rates to immune checkpoint therapies are in the range of 15 to 30% in most solid tumors, although they can reach as high as 45 to 60% in melanoma and tumors with high microsatellite instability (MSI-H) (28). For tumors that fail to respond to these therapies, CAR T cell therapy, which does not require pre-existing tumor-reactive T cells, is a promising treatment option to be discussed in the following sections.

## 2 Development of chimeric antigen receptor-engineered T cell therapy

### 2.1 The emergence of chimeric T cell receptor

The concept of a chimeric T cell receptor, which combines antibody-derived variable regions (VH/VL) with T cell receptor (TCR)-derived constant regions, was first reported in 1987 by a Japanese immunologist Dr. Yoshikazu Kurosawa and his team at the Institute for Comprehensive Medical Science in Aichi, Japan (29). This landmark study showed that the expression of anti-phosphorylcholine chimeric receptors in murine T-cell lymphoma EL4 cells resulted in calcium influx when challenged with phosphorylcholine-positive bacteria, suggesting the chimeric receptor could activate T cells in response to antigens (29).

Two years later, in 1989, Israeli immunologist Dr. Zelig Eshhar and his colleagues at the Weizmann Institute of Science described a similar approach to redirect T cells to recognize antigens in a non-major histocompatibility complex (MHC)-restricted manner (30). The resultant chimeric T-cell receptor (cTCR) was comprised of the anti-2,4,6-trinitrophenyl (TNP) antibody Sp6’s variable heavy and light chains, which were fused with the constant regions of the alpha and beta TCR chains, respectively. Upon co-transfection into murine MD.45 cytotoxic T lymphocyte hybridoma cells, the functional cTCRs were expressed on cell surface and able to bind to TNP antigen, leading to T cell activation as evidenced by interleukin-2 (IL-2) production and the killing of target cells. The MHC-independent activation of cTCR-expressing T lymphocytes was further demonstrated by IL-2 production upon binding to TNP-coupled proteins adsorbed onto a plastic substrate.

The double-chain heterodimeric cTCRs required infecting T cells with two separate retroviral vectors, leading to low co-transduction efficiency. To address this issue, Dr. Eshhar’s team designed a single-chain chimeric receptor in which the single-chain variable fragment (scFv) was fused to a lymphocyte intracellular signaling domain from either CD3ζ or FcϵRIγ. This resulted in the scFvR (31), also known as the first-generation CAR. The scFv antigen-binding domain was derived from a monoclonal antibody and retained the antigen-binding affinity and specificity of the parental antibody (32, 33). When expressed in MD.45 T-cell hybridoma cells, the scFvR conferred non-MHC-restricted activation upon encountering the antigen (31). Compared to cTCR, the scFvR had increased vector transduction efficiency and could independently transduce the T cell activation signal, bypassing the need for the conventional TCR complex. The double-chain cTCR and the single-chain scFvR were referred to as “T-bodies” (34) and are the prototypes of modern CAR.

### 2.2 The first-generation CAR

The first-generation CAR contains scFv fused to CD3ζ or FcϵRIγ and these engineered T cells showed anti-cancer activity in murine models. For example, an scFvR that contained anti-ERBB2/HER2 scFv and a murine CD3ζ signaling endodomain slowed the growth of subcutaneous tumors in athymic BALB/c mice (35). In another study, murine tumor-infiltrating T cells expressing a scFvR known as *MOv-γ*, which consisted of anti-α-folate receptor (FR) scFv and the Fc receptor γ chain, showed anticancer activity *in vitro*, in athymic nude mice, and in syngeneic C57BL/6 mice (36, 37). It is important to note that in these *in vivo* experiments, mice received systemic high-dose IL-2 treatment after the gene-modified T cells were infused (35, 37).

Based on the encouraging anticancer results *in vitro* and in mice (36–38), the first two clinical trials of CAR T cell therapy in humans were conducted in ovarian cancer patients using autologous T cells modified to express the chimeric receptor *MOv-γ* (39), and in metastatic renal cell carcinoma patients with autologous T cells expressing chimeric receptor scFv(G250) (40). However, no reduction in tumor burden was observed in any of the patients, and the genetically modified T cells quickly declined to undetectable levels in most patients within one to two months (39, 40), indicating that the lack of *in vivo* persistence may have contributed to the ineffectiveness of the infused cells. In another human trial, autologous T cells were transiently transfected with a chimeric receptor consisting of anti-CD20 scFv and human CD3ζ signaling endodomain. The engineered T cells showed persistence *in vivo* for up to 9 weeks and had limited anti-cancer activity(41). Similarly, in a phase 1 trial in children with recurrent neuroblastoma, engineered T cells that express CE7R(huCD3ζ) were persistent *in vivo* for up to 6 weeks (42). It is worth noting that in the last two trials, engineered T cells also expressed drug-selection genes, which could be immunogenic and impair the *in vivo* persistence of the engineered T cells (41, 42). The first-generation GD2-specific CAR T cells based on Epstein-Barr virus (EBV)-specific cytotoxic T cells showed certain persistence and antitumor activity in patients with neuroblastoma (43). However, the overall ineffectiveness of first-generation CARs in combating cancer in humans has led to efforts to optimize their design.

### 2.3 The second-generation CAR

T cell activation typically requires two signals: the first signal is triggered by the engagement of the TCR with peptide-loaded major histocompatibility complex (pMHC), and the second signal is provided by costimulatory receptors such as CD28 (44). It was therefore proposed that incorporating a costimulatory endodomain into engineered T cells could enhance their proliferation and persistence. Dr. Michel Sadelain’s laboratory at Memorial Sloan Kettering Cancer Center (MSKCC) designed a chimeric receptor that combines the CD3ζ and CD28 endodomains, which provides both activation and co-stimulatory signals, and leads to enhanced antigen-dependent proliferation, interleukin-2 production, and cancer cell killing *in vitro* (45, 46). Furthermore, T cells expressing chimeric receptors containing both CD3ζ and CD28 endodomains showed significantly increased expansion and persistence in human patients compared to T cells expressing chimeric receptors containing only the CD3ζ endodomain (47). These results clearly demonstrated the importance of including a costimulatory endodomain such as CD28 in the chimeric receptor.

**Figure 1:**
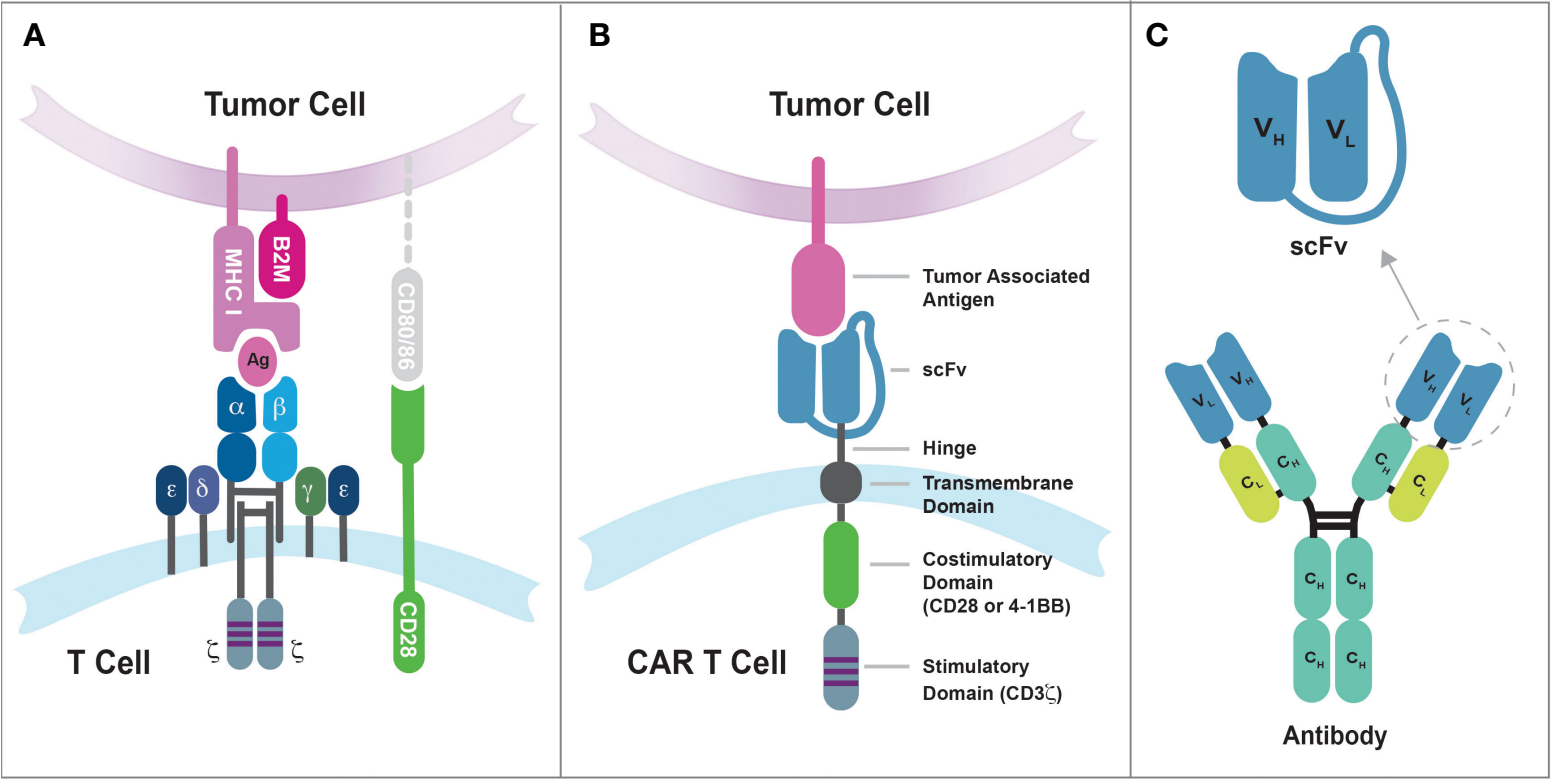
The differences in tumor antigen recognition between conventional TCRs and synthetic CARs. **(A)** TCR recognition of tumor antigens is restricted by MHC complex molecules, and the suboptimal efficiency of cancer cell killing by conventional T cells may be due in part to the lack of CD80/86 expression on tumor cells. The CD8 coreceptor has been omitted from the illustration for purpose of simplicity. **(B)** Synthetic CARs recognize tumor-associated antigens on the surface of cancer cells through the single-chain variable fragment (scFv) domain. These interactions then simultaneously activate both the CD3-mediated primary signal and the CD28/4-1BB-mediated secondary signal in T cells. **(C)** The scFv domain is derived from a monoclonal antibody and consists of the variable regions from the heavy chain (V_H_) and light chain (V_L_) linked by a flexible linker sequence.

The incorporation of the 4-1BB/CD137 signal transduction domain in the CAR design, pioneered by Dr. Dario Campana at St Jude Children’s Research Hospital, represents another significant advancement in the field (48–50). The inclusion of 4-1BB costimulatory endodomain significantly improved the persistence and antitumor activity of CAR-engineered T cells in mice (50). Moreover, using the elongation factor-1α promoter (EF-1α) in lentiviral vector has resulted in more stable and long-term expression of chimeric receptors in T cells compared to other promoters from cytomegalovirus (CMV), phosphoglycerate kinase (PGK), and ubiquitin (50). The CAR construct that incorporates both a costimulatory endodomain (such as CD28 or 4-1BB) and the CD3ζ signaling endodomain is classified as a second-generation CAR and later achieved remarkable success in human clinical trials, marking a significant milestone in the development of CAR T cell therapy. **Figure 1** illustrates the comparison between the conventional TCR recognizing peptide-major histocompatibility complex (pMHC) and the second-generation CAR recognizing tumor-associated antigen. Additionally, it shows how the single-chain variable fragment (scFv) domain is derived from a monoclonal antibody.

### 2.4 The clinical success of CAR T cell therapy

The second-generation CAR T cell therapy demonstrated effectiveness in one patient with advanced follicular lymphoma at NCI (51), and in patients with refractory CLL and relapsed B-cell ALL at MSKCC (52). The therapy at NCI involved the use of a retroviral vector called MSGV to express a CD19-specific CAR. This CAR was designed to target CD19, a protein found on the surface of B-lineage cells, using an anti-CD19 scFv derived from FMC63 murine monoclonal antibody. It contained a CD28 costimulatory endodomain and a CD3ζ endodomain. The patient received lymphodepletion followed by two doses of CAR T cells, along with eight doses of IL-2. As a result of this treatment, the patient achieved partial remission of the lymphoma and selective elimination of B-lineage cells(51). In the MSKCC Phase 1 trial, autologous CD19-targeted CAR T cells expressing the second-generation CAR (19-28z) were evaluated for their safety and persistence in treating relapsed or chemotherapy refractory CLL and B-ALL. Patients who received prior conditioning with cyclophosphamide exhibited a partial response, whereas patients treated without prior conditioning did not show any objective disease responses (52).

A critical breakthrough in the use of CAR T cell therapy arrived when Dr. Carl June’s team at the University of Pennsylvania reported that three adult patients with advanced chronic lymphocytic leukemia (CLL) achieved complete or partial remission after receiving CD19-specific CAR T cell therapy (53, 54). The CD19-CAR construct used in this trial contained an anti-CD19 scFv (derived from FMC63), a 4-1BB costimulatory endodomain, and a CD3ζ signaling endodomain. It was expressed from a lentiviral vector driven by the EF1-α promoter. Upon infusion, the CAR T cell underwent significant expansion in patients, increasing in number by up to 1,000 times. These results unlocked the potential of the second-generation CAR T cell therapy to effectively treat advanced cases of CLL and other B-cell malignancies.

The results of these clinical trials confirmed that preparative lymphodepletion, a type of chemotherapy that reduces the number of immune cells in the body, is essential for the success of CAR T cell therapy. In contrast, the use of IL-2 does not seem to be necessary. Lymphodepletion was first shown to be effective by Dr. Steven Rosenberg’s team, who demonstrated that administering a combination of cyclophosphamide and fludarabine as a lymphodepleting chemotherapy led to the *in vivo* proliferation and migration of infused tumor-reactive T cells to tumor sites (22, 55, 56). Lymphodepletion may work by reducing the presence of endogenous lymphocytes that compete with infused T cells, while also increasing the levels of T cell growth factor, such as IL-15, in circulation (57). This would enable the infused T cells to expand more effectively in the body.

### 2.5 FDA approval of CAR T cell therapies for hematological cancers

**Figure 2:**
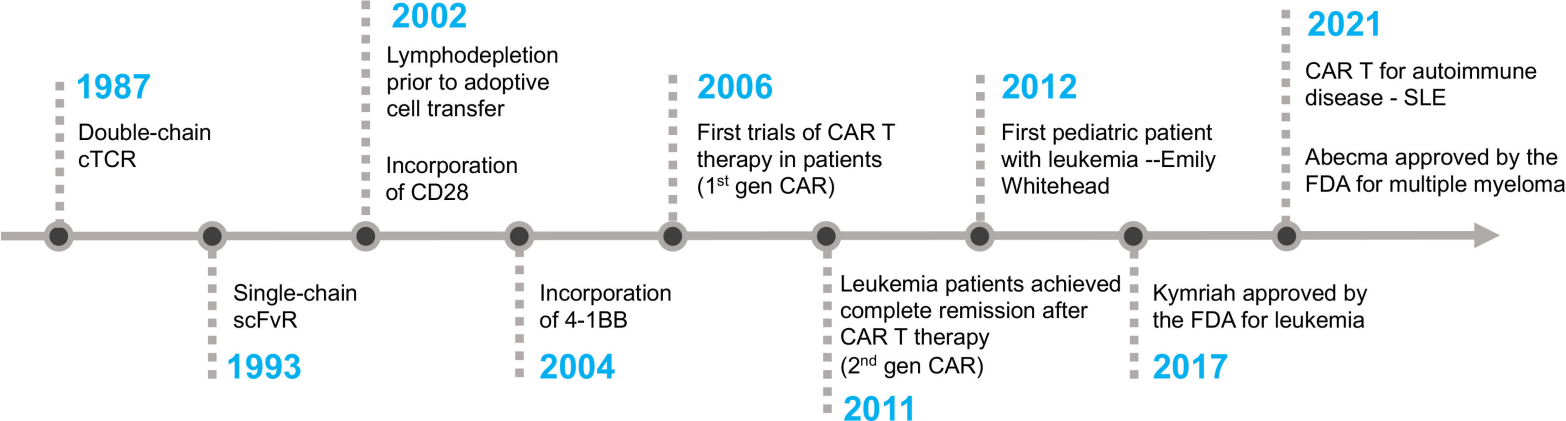
The timeline of key milestones in the development of CAR T cell therapy.

The first CAR T cell therapy approved by the FDA was tisagenlecleucel (Kymriah) on Aug 30^th^, 2017, for the treatment of pediatric and young adult Acute Lymphoblastic Leukemia (58). It was manufactured by Novartis Pharmaceuticals corporation. Later, three more CD19-specific CAR T cells were approved by the FDA for the treatment of different B cell malignancies namely axicabtagene ciloleucel (Yescarta), brexucabtagene autoleucel (Tecartus), and lisocabtagene maraleucel (Breyanzi) (59–61). The CAR expressed in Tecartus is identical to that in Yescarta, but their manufacturing processes differ as Tecartus involves T cell enrichment while Yescarta does not (62). In April 2021 and February 2022, two BCMA-specific CAR T cell therapies were approved for the treatment of multiple myeloma, namely idecabtagene vicleucel (Abecma) and ciltacabtagene autoleucel (Carvykti) (63, 64). **Table 1** provides detailed information on each of the FDA-approved CAR T cell-based therapies. **Figure 2** depicts the key milestones in the development of CAR T cell therapy.

**Table 1:** Summary of FDA-approved CAR T cell therapies for B cell malignancies and multiple myeloma.

Company name Brand name Generic name	Date of approval	Target antigen/ Antibody	Hinge/ transmembrane	Costimulatory domains	Vector/ promoter	Targeted cancers	Pivotal trial	No. of Patients	Outcomes	References
Novartis Kymriah Tisagenlecleucel	Aug 30, 2017	CD19 Mouse FMC63	CD8α/CD8α	4-1BB + CD3ζ	Lentiviral EF1α	R/R CAYA B-ALL	ELIANA (NCT02228096)	75	81% overall remission rate	(58)
Kite Yescarta Axicabtagene ciloleucel	Oct 18, 2017	CD19 Mouse FMC63	CD8α/CD8α	CD28 + CD3ζ	Gammaretroviral LTR	R/R LBCL	ZUMA-1 (NCT02348216)	108	58% complete response	(59)
Kite Tecartus Brexucabtagene autoleucel	Jul 24, 2020	CD19 Mouse FMC63	CD28/CD28	CD28 + CD3ζ	Gammaretroviral LTR	R/R MCL	ZUMA-2 (NCT02601313)	68	67% complete response	(61)
Juno Breyanzi Lisocabtagene maraleucel	Feb 5, 2021	CD19 Mouse FMC63	IgG4/CD28	4-1BB+ CD3ζ	Lentiviral EF1α	R/R LBCL	Transcend NHL001 (NCT02631044)	269	53% complete response	(60)
Bluebird Abecma Idecabtagene vicleucel	Mar 26, 2021	BCMA Mouse BB2121	CD8α/CD8α	4-1BB+ CD3ζ	Lentiviral MND	R/R MM	KarMMa (NCT03361748)	128	33% complete response	(63)
J&J and Legend Carvykti Ciltacabtagene autoleucel	Feb 28, 2022	BCMA dual camel single-domain antibodies	CD8α/CD8α	4-1BB + CD3ζ	Lentiviral EF1α	R/R MM	CARTITUDE-1 (NCT03548207)	97	82.5% complete response	(64)

R/R, relapsed or refractory. CAYA, children and young adults. LBCL, large B-cell lymphoma. MCL, mantle cell lymphoma. MM, multiple myeloma.

William Paul Ludwig was the first adult patient to receive CAR T cell therapy at the University of Pennsylvania. He was suffering from refractory chronic lymphocytic leukemia (CLL). After receiving the therapy in 2010, he was in complete remission for more than 10 years until his passing in early 2021 due to COVID-19 pneumonia. Emily Whitehead made history as the first pediatric patient with B-ALL leukemia to receive CD19-CAR T cell therapy in April 2012. She developed severe cytokine release syndrome (CRS) and acute respiratory distress syndrome (ARDS) as side effects of the therapy. Dr. Carl June’s team ingeniously employed tocilizumab, an anti-IL6 receptor antibody initially approved for rheumatoid arthritis, to promptly and effectively alleviate the severe side effects of CRS (65). Eleven years after her treatment, Emily remains cancer-free, despite being given only a few weeks to live prior to her CAR T cell therapy. Emily’s story is a testament to the groundbreaking advancements in CAR T cell therapy and the unwavering dedication of visionary medical professionals who have pushed the boundaries of medical science.

While CD19-CAR T cell therapy has shown a high response rate in children and young adults with B-ALL, the majority of these patients eventually experience relapse with CD19-negative disease. Consolidative allogeneic hematopoietic stem-cell transplant (alloHSCT) can improve durable disease control (66). CD22-targeted CAR T cell therapy has demonstrated high efficacy in treating B-ALL, whether the disease is naïve to or resistant to CD19-CAR T cell therapy (67). Bispecific CD19/CD22 CAR T cell therapy combined with alloHSCT has shown efficacy and favorable outcomes in pediatric and young adult patients with R/R B-ALL (68). In adult patients with B-ALL, a high disease burden is a significant predictor for disease progression after CD19-CAR T Cell therapy (69). Approximately 40% of relapsed patients have responded to salvage treatments, such as chemotherapy or a second infusion of CAR T cells, but remission duration has been short, even with consolidative alloHSCT.

Ironically, despite being one of the first diseases treated with CAR T cell therapy, CLL has not yet been approved for CAR T cell therapy by the FDA. The overall response rate of CD19-CAR T cell therapy for CLL is approximately 30%, which is low compared to other B-cell malignancies. This relative inefficiency may be attributed to T cell exhaustion in CLL patients (70).

In addition, CAR T cell therapy faces unique challenges in treating T-cell malignancies, including fratricide, T-cell aplasia, and production contamination with malignant T cells. T cell differentiation markers such as CD5, CD7, CD3 have been tested as targets for CAR T cell therapy. Interestingly, targeting CD5 appears to have only minor fratricidal issues, due to the downregulation of CD5 from the surface of CAR T cells(71). Another strategy is to selectively target malignant T cells through one of the two TCRβ-chain constant regions (TRBCs) or one of the 30 TCRβ-chain variable regions (TRBVs), thereby preserving normal T cells that do not utilize the specific TRBC or Vβ found in malignant T cells (72, 73).

Long-term follow-up studies have shown the potential of CAR T cells as a living drug. Specifically, a study on two adult patients (Ludwig and Olson) ten years post CD19 CAR T cell infusion demonstrated two different phases of CAR T cellular response *in vivo* (74). During the initial phase, CD8^+^ CAR T cells were the major cell population responsible for targeting the malignant cells. However, their numbers eventually declined. In the second phase, which occurred in the long term, over 95% of CAR T cells are proliferative CD4+ T cells, which might have contributed to the sustained remission of chronic lymphoid leukemia. In another long-term follow-up study, 43 patients with relapsed B-cell malignancies received anti-CD19 CAR T cell therapy (Axicabtagene ciloleucel; FMC63-28z). 51% of them  showed a complete remission lasting ≥3 years, with late adverse events being rare (75). These findings demonstrate the potential of CAR T cell therapy as a long-lasting therapeutic option for the treatment of malignancies.

A meta-analysis of 38 reports, including 2,134 patients with relapsed or refractory acute lymphoblastic leukemia (RR/B-ALL), found that the use of 4-1BB costimulatory endodomain, low-dose cyclophosphamide lymphodepletion, and pretreatment morphologic remission were associated with favorable overall survival (76). These results suggest that incorporating these factors into future treatment strategies may improve outcomes for patients with RR/B-ALL. In a separate retrospective analysis of 809 patients with R/R DLBCL, treatment with Axicabtagene-ciloleucel, which uses the CD28 costimulatory endodomain in a gammaretroviral vector, was found to have greater efficacy but also a higher incidence of toxicity when compared to Tisagenlecleucel, which uses the 4-1BB costimulatory endodomain in a lentiviral vector (77).

## 3 Major challenges to overcome for CAR T cell therapy

### 3.1 Severe adverse events

CAR T cell therapy has shown great promise in the treatment of hematological cancers, but one major concern with this approach is the potential for life-threatening adverse events. Two of the most common adverse events are cytokine release syndrome (CRS) and immune effector cell-associated neurotoxicity syndrome (ICANS). CRS is mediated by the cytokines IL-1 and IL-6, which can cause fever, hypotension, and other systemic symptoms. To mitigate the risk of CRS and neurotoxicity, the FDA approved the use of the humanized anti-IL6 receptor antibody tocilizumab in 2017 for CAR T cell therapy. Other potential treatments for these adverse events include the IL-1 receptor antagonist anakinra and the anti-IL6 chimeric antibody siltuximab (78, 79). The underlying mechanisms of CRS and ICANS are complex. In studies conducted on murine models of CRS, it was found that monocytes or macrophages were the primary source of IL-1 and IL-6 (80, 81). Notably, CRS was prevented when monocytes were depleted or IL-6 receptors blocked, and both CRS and neurotoxicity were abolished by the use of the IL-1 receptor antagonist, anakinra (81). Another study shows that CD19, a protein commonly targeted by CAR T cell therapy in B cell malignancies, is also expressed in the brain mural cells. This raises the possibility of an on-target off-tumor mechanism for CD19-CAR T cell therapy-associated neurotoxicity (82).

The severity of adverse events associated with CAR T cell therapy is influenced by several factors, including the pretreatment tumor burden, lymphodepletion regimen intensity, and CAR T cell dose (83). Elevated cytokine levels during CD19-CAR T cell therapy have been linked to pretreatment tumor burden(84, 85). While tumor burden does not appear to affect CD19-CAR T cell expansion peaks, it can negatively affect the complete remission rates and possibly overall survival (86).

The association between the severity of adverse events and disease burden is supported by the application of CD19-CAR T cell therapy in autoimmune diseases. In a small series of human trials of CD19-CAR T cell therapy for systemic lupus erythematosus (SLE), patients who received autologous CAR T cell therapy experienced significant *in vivo* CAR T cell expansion, swift alleviation of lupus symptoms, but little to no adverse events (87, 88). Similarly, a 41-year-old man with the refractory antisynthetase syndrome (ASyS) experienced minimal adverse events after receiving CD19-CAR T cell therapy, despite the significant expansion of CD19-CAR T cells *in vivo* (89).

While patients with CLL and B-cell ALL have markedly elevated B cell counts in peripheral blood (90, 91), patients with autoimmune diseases such as SLE or AsyS have reduced or unchanged B cell counts (92–94). The lack of serious adverse events in SLE or AsyS patients who have undergone CAR T cell therapy suggests that the severe toxicities experienced by cancer patients are not directly caused by the CAR T cells themselves. Rather, they appear to be related to the massive destruction of tumor cells by CAR T cells, which can result in the release of intracellular contents into the bloodstream and trigger tumor lysis syndrome (TLS), a potentially life-threatening condition (95, 96). This finding raises the possibility of preventing severe adverse events by treating cancer patients with CAR T cell therapy at the early stages of metastasis when the tumor burden is low.

### 3.2 High cost of manufacturing autologous CAR T cells

One limitation of current CAR T cell therapy is the high cost of manufacturing autologous CAR T cells, which can result in a total treatment cost of up to $500,000 for patients with severe CRS (97). Additionally, the conventional turnaround time for autologous CAR T cell manufacturing varies from 21 to 35 days. During this waiting period, patients may require bridging therapy and, in some cases, succumb to rapidly advancing disease without benefiting from CAR T cell therapy. Furthermore, T cells from sick patients may suffer from exhaustion and be less active than T cells from healthy donors. Therefore, to make this treatment more affordable and readily available, several strategies are being tested, including the use of off-the-shelf allogenic CAR (allo-CAR)T cells and *in vivo* generation of CAR T cells.

When administering allogeneic CAR T cells to patients, there is a risk of graft-vs-host disease (GvHD) and CAR T cell rejection by the host immune system. To mitigate this risk, the TCR genes are deleted from the CAR T cells using the CRISPR/Cas9 system to prevent GvHD. Additionally, the formation of the HLA class I complex is abolished by deleting the beta-2-microglobulin gene (B2M) to avoid host rejection of CAR T cells (98). However, a significant challenge in allogenic CAR T cell therapy is that allo-CAR T cells do not survive for long periods in patients, thereby compromising the therapy’s anticancer efficacy (99).

There are several explanations for the reduced persistence of allo-CAR T cells *in vivo*. One possibility is that allo-CAR T cells that do not express HLA class I complex may be targeted and eliminated by recipient natural killer cells through “missing-self” mechanisms. HLA class I complex may also play a role in the survival of mature T cells *in vivo*, as in B2M knockout mice, CD8^+^ T cells are completely missing (100). Another factor is that the CRISPR/Cas9 system can create unintended genetic rearrangements such as large chromosome deletion or even karyotypic abnormalities. An alternative genome editing technology, base editing, does not generate DNA double-stranded breaks (DSBs) and may induce less genomic damage to the T cells. Base-edited allo-CAR T cells were used to treat refractory T-cell leukemia (T-ALL) (101, 102). However, in this trial, B2M was not deleted and therefore HLA class I complex is still intact in allo-CAR T cells (101). In addition, TALEN-mediate gene editing is also used to disrupt endogenous TRAC and B2M loci to generate immune-evasive universal CAR T cell therapy (103).

Another interesting strategy to reduce cost is to generate CAR T cells *in vivo*. Transient CAR T cells can be generated *in vivo* by delivering mRNA encoding the FAP-targeting CAR in lipid nanoparticles (LNPs) (104). By direct intravenous infusion of replication-incompetent VSV-G-pseudotyped lentiviral particles encoding a CD19-targeting CAR transgene, persistent CAR-transduced CD3^+^ T cells were produced and complete B cell aplasia was achieved in mice (105). With this approach, CAR-encoding lentiviruses are directly infused into the patient and viral transduction of T cells occurs spontaneously *in vivo*. Generation of CAR T cells *in vivo* would eliminate the need for *ex vivo* manufacturing of the engineered cells, and therefore significantly reduce the cost of CAR T cell therapy.

Moreover, shortening the autologous CAR T cell manufacturing process can also lower costs. The typical process includes T cell activation, viral transduction, and *ex vivo* expansion for at least one week. The *ex vivo* expansion of huCART19-IL18, the humanized anti-CD19 CAR co-expressed with IL-18, is shortened to 3 days and still produces CAR T Cell with encouraging early efficacy in a Phase 1 trial (106). Another study has shown that CAR T cells can be generated within 24 hours through the transduction of non-activated T cells without additional expansion and still exhibit anti-cancer efficacy in mice (107).

### 3.3 Ineffectiveness against solid tumors

Solid tumors represent approximately 90% of human cancers in adults and 30% in children. Despite unprecedented success in hematological cancers, CAR T cell therapy has been far less impressive in solid tumors. There are two major challenges for solid tumors, including immunosuppressive tumor microenvironment (TME) and lack of tumor-exclusive target.

TME is enriched with regulatory T cells, tumor-associated macrophages (TAMs), and CD4+ T helper 2 (Th2) cells, which produce anti-inflammatory cytokines such as IL-10, TGF-β, and IL-4. To overcome the immunosuppressive TME, CAR T cells are engineered to express various accessory molecules, called “armored” CAR, that can enhance tumor infiltration and increase the CAR T cells’ antitumor activity by reducing the immunosuppression in the tumor microenvironment. To date, numerous “armored” CAR constructs have been reported in the literature to express accessory molecules, including cytokines such as IL7, IL12, IL-15, IL-18, and IL-21, as well as other molecules like dominant negative TGFβ receptor, constitutively signaling IL-7 receptor C7R, noncoding RNA RN7SL1,c-Jun, CD40L, BATF, and PRODH2 (108–112). IL-2 is a potent stimulator of T cell proliferation; however, systemic administration of IL-2 has serious side effects. To bypass the toxicity, one strategy involves engineering CAR T cells to express a second receptor called synNotch, which can secrete IL-2. This might promote CAR T cell proliferation in the suppressive tumor microenvironment (113).

In addition to co-express a stimulatory factor, negative regulators of T cell function are being deleted from CAR T cells by CRISPR/Cas9 technology to achieve an enhanced anticancer activity, including PD-1 (114, 115), Tet2 (116), NR4A (117), and regnase-1 (118, 119).

The “armored” CAR T cell therapies have demonstrated superior anticancer activity against solid tumors in preclinical mouse models. To determine whether this translates to clinical applications, they must undergo rigorous human trials. However, a critical concern is that the delicate equilibrium between positive and negative signaling in T cells is crucial for preserving immune homeostasis and preventing autoimmunity and inflammation. Permanent disruption of negative regulators or constitutive amplification of positive signals could upset the balance and result in unintended consequences.

The lack of a tumor-exclusive membrane target presents another significant challenge, potentially the most significant challenge, for solid tumors. The ideal target for CAR T cell therapy would be a membrane protein or glycolipid that is expressed solely on the surface of cancer cells and not in any normal tissues. However, in theory, such a target does not exist because any protein lacking normal tissue function would not have been conserved during evolution. Currently, several targets for solid tumors are in various stages of clinical development, including GD2, HER2, EGFRvIII, Mesothelin, Claudin-18.2, IL13Ra2, CEA, PSMA, PSCA, GPC3, MUC1, among others (120). While highly expressed in solid tumors, these targets, except for EGFRvIII, are also present in certain normal tissues. Even CD19, the successful target for CAR T cell therapy in B-cell malignancies, is expressed in normal B cells. Consequently, patients receiving CD19-CAR T cell therapy develop B cell lymphopenia and are more susceptible to infections, even with immunoglobin replacement therapy (121). One way to enhance the specificity of CAR T cell therapy is to engineer T cells to express dual CARs that recognize two distinct antigens present on the same cancer cells (122, 123). This approach can selectively target and eliminate only cancer cells expressing both antigens while minimizing damage to healthy cells that express one antigen.

Numerous clinical trials are currently underway to test CAR T cell therapy in solid tumors. Among them, GD2-CAR T cell therapy has shown promising results. GD2 is a disialoganglioside and expressed in normal neural tissues, such as the cerebellum and peripheral nerves in humans, and is highly expressed in tumors of neuroectodermal origin, including neuroblastoma and diffuse intrinsic pontine glioma (DIPG). In a Phase 1 trial involving four patients with H3K27M-mutated DIPG, patients received intravenous CAR T cell infusion followed by intracerebroventricular infusion. Three out of four patients showed clinical improvement, and interestingly, no signs of on-target, off-tumor toxicity were observed (124). In another Phase 1-2 clinical trial in relapsed or refractory high-risk neuroblastoma, GD2-CAR T cell therapy demonstrated impressive efficacy, with an overall response rate of 63% and a complete response rate of 33% (125). These results suggest that GD2-CAR T cell therapy is feasible and safe for treating high-risk DPIG and neuroblastoma.

It is noteworthy that in the DIPG trial, the GD2-CAR contains two signaling domains, 4-1BB and CD3ζ, whereas in the neuroblastoma trial, the GD2-CAR contains three signaling domains, including CD28, 4-1BB, and CD3ζ. The scFv domain in both CARs was derived from the same anti-GD2 murine antibody 14g2a, which has a relative low affinity to GD2 (KD=77 ± 8 nM) (126). These encouraging results from GD2-CAR T cell therapy in two types of solid tumors indicate that even if the target is expressed in certain normal tissues, anticancer efficacy can be achieved in solid tumors with manageable toxicity. The lack of significant toxicity in GD2-CAR T cell therapy could be attributed to the observation that CAR T cells requires high antigen density for full effector function (127).

Furthermore, as CAR T cells are a living drug, any persistent CAR T cells in the body will continue to attack normal tissues expressing the target. To avoid this, one strategy is to eliminate the CAR T cells from the body after curing cancer. The incorporation of a safety switch to the CAR construct would potentially achieve this.

## 4 Safety switch and controllable CAR

Gene and cell therapy are constantly evolving, and safety remains a paramount concern in their development. One potential risk is therapy-induced tumorigenesis, where the therapeutic gene insertions lead to unintended consequences, such as activating oncogenes or disrupting tumor suppressor genes, resulting in the development of insertional mutagenesis. So far, the use of lentiviral or γ-retroviral vectors in CAR T cell therapy has shown high safety, with no reported cases of transformation in about 20,000 treated patients. However, a Phase 1 clinical trial using piggyBac transposon-based CD19-CAR T cell therapy raised concerns after 2 out of 10 patients developed malignant CAR T cell-derived lymphoma within 12 months post-infusion (128, 129). These findings highlight the need for a safety switch that can eliminate CAR T cells whenever necessary.

In addition, CAR T cells are a living drug that can potentially persist in the body for an extended period after being infused into patients. Because antigens targeted by the CAR T cells are usually also expressed in some healthy tissues, the long-term persistence of CAR T cells could present a safety risk by attacking the normal tissues. One solution is the incorporation of a safety switch in the CAR design, which would allow for the controlled removal of the CAR T cells if necessary.

There are several safety switches reported in the literature. The inducible caspase 9 (iC9)/Rimiducid has demonstrated its effectiveness in eliminating CAR T cells both *in vitro* and in mice (130). The dimerizing drug Rimiducid has no observable toxicity and is very well tolerated in humans (131) and demonstrated high efficacy in removing CD19-CAR T cells in patients (132). A rapamycin-induced caspase 9 is an alternative safety switch with a similar suicidal mechanism (133). CD20 full-length protein or mimotope can also be used as an effective safety switch, and anti-CD20 antibody Rituximab can quickly eliminate engineered T cells that express CD20 (134–137).

In addition to “off” switches, there are also “on” switches that can be used to control CAR T cell activity. Examples of such controllable CAR T cell therapies include a Rimiducid-inducible GoCAR-T, a small molecule-gated CAR, synZiFTR-regulated CAR, and protease inhibitor-regulated SNIP CAR (138–141). The tyrosine kinase inhibitor dasatinib was reported as a reversible pharmacologic switch (142, 143).

In summary, a safety switch or controllable CAR can serve several purposes. Firstly, during treatment, CAR T cells can be quickly depleted or turned off in the face of life-threatening toxicity when corticosteroids and tocilizumab fail to control adverse events (132). Secondly, after cancer is cured, CAR T cells can be removed to avoid long-term side effects caused by the killing of normal tissues that express targets. This is particularly important in cases where the on-target, off-tumor toxicity is almost inevitable due to the absence of tumor-specific targets in solid tumors. Thirdly, although CAR T-lymphoma is rare, a built-in safety switch can be used to kill the CAR T-lymphoma if it occurs.

## 5 CAR T cell therapy beyond cancer

CAR T cell therapy is not limited to cancer treatment and is being explored for the treatment of various pathological conditions such as autoimmune diseases, fibrotic diseases, infectious diseases, etc. The first application of CAR T cell therapy was in the treatment of HIV using the CD4ζ-CAR (144). This pioneering study demonstrated the safety and long-term persistence of retroviral-modified T cells in patients (145). Most recently, CD19-CAR T cell therapy has shown remarkable results in the treatment of systemic lupus erythematosus and antisynthetase syndrome (87–89). This therapy has proven to be highly effective in alleviating symptoms in both autoimmune diseases, with minimal adverse events reported.

Fibrotic diseases like cardiac and liver fibrosis are also being targeted with CAR T cell therapy. The fibroblast activated protein (FAP)-targeted CAR T cell therapy significantly reduced pathological cardiac fibrosis and restored function after injury in mice (146). Similarly, a senolytic CAR T cell therapy, which targets the urokinase-type plasminogen activator receptor (uPAR), successfully reversed senescence-associated pathologies, including liver fibrosis in mice (147). Infections caused by human immunodeficiency (HIV), Hepatitis B and C viruses, and human cytomegalovirus may also be treated with CAR T cell therapy targeting specific viral proteins expressed on the host cells (148).

## 6 Future perspectives

The remarkable achievement of CAR T cell therapy has inspired scientists to explore the potential of engineering other immune cells, such as natural killer (NK) cells, NKT cells (149), macrophages (150), and neutrophils (151), for therapeutic purposes. Among these, CAR-NK cell therapy has shown impressive responses in human clinical trials (152). While these immune cells may have fewer concerns of graft-vs-host disease, which could make them more suitable as off-the-shelf products, they also have their own limitations, including short life spans, limited proliferation capabilities, and inability to form memory cells. Additionally, T cells can be engineered to target tumors through tumor-neoantigen-specific TCRs (153). TCR-T cell therapy has a significant advantage in that the target is not limited to membrane antigens, although elegantly designed CAR can also recognize intracellular neoantigens in the context of MHC complexes (154).

In conclusion, CAR T cell therapy has made significant progress in the treatment of cancer, but there are several challenges that need to be overcome to make this treatment widely available and effective. Ongoing research in the development of CAR T cell therapy for solid tumors, off-the-shelf CAR T cell therapy, safety, cost, and non-cancer diseases will be critical to the future success of this treatment. The progress made in CAR T cell therapy highlights the importance of continued investment in scientific research and innovation. We look forward to a future where CAR T cell therapy and other immunotherapies are widely available and effective in treating cancer and other diseases, improving the lives of patients worldwide.

## Author contributions

BH and QF conceived the idea for the review article. AM and AB conducted the literature search and drafted the initial manuscript. SG and LH provided critical feedback and helped to revise the manuscript. BH and QF contributed to the writing and editing of the final manuscript. All authors contributed to the article and approved the submitted version.
